# Using Dynamic Contrast-Enhanced Magnetic Resonance Imaging Data to Constrain a Positron Emission Tomography Kinetic Model: Theory and Simulations

**DOI:** 10.1155/2013/576470

**Published:** 2013-10-03

**Authors:** Jacob U. Fluckiger, Xia Li, Jennifer G. Whisenant, Todd E. Peterson, John C. Gore, Thomas E. Yankeelov

**Affiliations:** ^1^Department of Radiology, Northwestern University, Chicago, IL 60611, USA; ^2^Institute of Imaging Science, Vanderbilt University, Nashville, TN 37212, USA; ^3^Department of Radiology and Radiological Sciences, Vanderbilt University, Nashville, TN 37212, USA; ^4^Program in Chemical and Physical Biology, Vanderbilt University, Nashville, TN 37212, USA; ^5^Department of Physics and Astronomy, Vanderbilt University, Nashville, TN 37212, USA; ^6^Department of Biomedical Engineering, Vanderbilt University, Nashville, TN 37212, USA; ^7^Department of Molecular Physiology and Biophysics, Vanderbilt University, Nashville, TN 37212, USA; ^8^Department of Cancer Biology, Vanderbilt University, Nashville, TN 37212, USA

## Abstract

We show how dynamic contrast-enhanced magnetic resonance imaging (DCE-MRI) data can constrain a compartmental model for analyzing dynamic positron emission tomography (PET) data. We first develop the theory that enables the use of DCE-MRI data to separate whole tissue time activity curves (TACs) available from dynamic PET data into individual TACs associated with the blood space, the extravascular-extracellular space (EES), and the extravascular-intracellular space (EIS). Then we simulate whole tissue TACs over a range of physiologically relevant kinetic parameter values and show that using appropriate DCE-MRI data can separate the PET TAC into the three components with accuracy that is noise dependent. The simulations show that accurate blood, EES, and EIS TACs can be obtained as evidenced by concordance correlation coefficients >0.9 between the true and estimated TACs. Additionally, provided that the estimated DCE-MRI parameters are within 10% of their true values, the errors in the PET kinetic parameters are within approximately 20% of their true values. The parameters returned by this approach may provide new information on the transport of a tracer in a variety of dynamic PET studies.

## 1. Introduction 

 There is an extensive literature on the use of compartmental modeling to understand the distribution and retention of various positron emission tomography (PET) radiotracers (see, e.g., [1, 2]). A series of ordinary, first-order, linear differential equations are often used to model the body as a series of well-mixed “compartments,” between which a tracer may be transported. Solving the differential equations and then fitting those solutions to measured tissue time activity curves (TACs) return estimates of a number of relevant physiological parameters. Typical dynamic PET models return parameters describing the metabolic rates of tracer utilization. The models used to extract these parameters have several free parameters and the measured TAC is, in practice, a weighted sum of unknown TACs from multiple compartments. This results in the introduction of extra assumptions into the analysis. Another central issue in standard dynamic PET modeling is the difficulty of obtaining a reasonable time course of the concentration of the injected tracer in the blood plasma (i.e., the arterial input function), especially for small animal studies. Thus, the current state-of-the-art in PET kinetic modeling typically requires simplifying assumptions to reduce the number of free parameters and/or nonlinear fitting methods which are well known to be sensitive to measurement noise [1]. Here, we introduce a method that exploits the data available from dynamic contrast-enhanced magnetic resonance imaging (DCE-MRI) studies to constrain the kinetic modeling of nuclear measurements and potentially provide new insight into the physical distribution and transfer of PET radiotracers.

 Similar to a kinetic PET study, DCE-MRI involves the serial acquisition of images before, during, and after the injection of a contrast agent [3]. As the contrast agent perfuses into the voxel (or region) of interest, it changes the tissue's native relaxation rates to an extent determined by the concentration of the contrast agent. By following the image sequence and fitting the resulting signal intensity with an appropriate mathematical model, various parameters related to blood vessel perfusion and permeability and tissue volume fractions (i.e., the blood fraction, extravascular-extracellular space (EES), and extravascular-intracellular space (EIS)) can be extracted. The three volume fraction parameters can potentially be used to constrain the modeling of kinetic PET data; that is, the formalism below allows the separation of the overall tissue TAC into TACs associated with the blood, EES, and EIS compartments. This enables access to compartments that are not typically accessible in dynamic PET studies.

 It is important to note that there exists a subtle, though fundamental, difference between the compartmental models employed in quantitative DCE-MRI and dynamic PET analyses. The compartments in kinetic PET modeling typically (^15^O labeled H_2_O is one notable exception) refer to biochemical compartments (e.g., bound or free), whereas the compartments in kinetic MRI modeling refer to physical compartments (e.g., the blood, EES, or EIS). Thus, when the compartments extracted from a DCE-MRI analysis are used to separate the PET TAC into different compartments, the TAC is separated into the compartments determined from the DCE-MRI data—and these compartments are fundamentally different than the biochemical compartments. This means that the TACs associated with these compartments, as well as the kinetic parameters describing the movement of the tracer between these compartments, are not the same as those reported in the existing PET literature. More specifically, in this contribution, we develop the formalism required to use DCE-MRI data to extract separate TACs for the blood pool (i.e., the input function), EES, and EIS and then show how these time courses can be used to fit simplified versions (i.e., fewer free parameters with known TACs) of a PET compartmental model to extract kinetic parameters related to the delivery and retention of PET tracer that is distributed amongst the blood space, EES, and EIS. We conclude by discussing how the access to these new physiological parameters may be of use in future dynamic PET studies.

## 2. Materials and Methods

### 2.1. PET Kinetic Modeling

**Figure 1:**
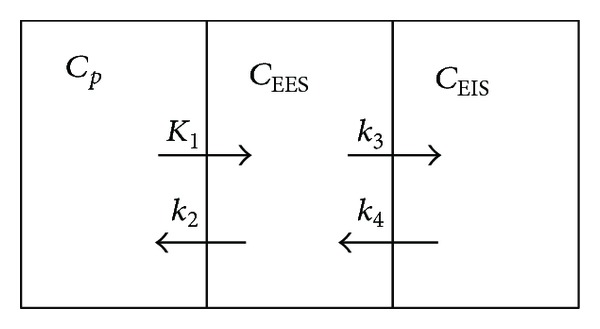
A schematic representation of the three-compartment model used with the dynamic PET imaging. From left to right, the three compartments represent the blood plasma, the extracellular-extravascular space, and the extracellular-intravascular space.

Figure 1 depicts the compartmental model that we will use for this study; from left to right, the compartments are the plasma, extravascular-extracellular space, and extravascular-intracellular space, respectively. We again note that these compartments are not those identified in a typical PET kinetic modeling, which consider the three (biochemical) compartments of radiotracer distribution as plasma, free and nonspecifically bound in tissue, and specifically bound [1]. Rather, the (physical) compartments chosen here reflect those typically identifiable in a dynamic contrast-enhanced MRI acquisition described below. The set of compartments described by this model provides access to other potentially useful compartments and rate constants. Thus, while the mathematical description of the tracer concentrations is unchanged, it does change the interpretation of the parameter values; we return to this important point in Section 4. The following set of first-order, ordinary, linear differential equations describe the system depicted in Figure 1:

(1) $$\begin{array}{c} \frac{{dC}_{p}\left(t\right)}{dt}=k_{2}C_{\text{EES}}\left(t\right)-K_{1}C_{p}\left(t\right)\text{,}\\ \frac{{dC}_{\text{EES}}\left(t\right)}{dt}=K_{1}C_{p}\left(t\right)-k_{2}C_{\text{EES}}\left(t\right)-k_{3}C_{\text{EES}}\left(t\right)+k_{4}C_{\text{EIS}}\left(t\right),\\ \frac{{dC}_{\text{EIS}}\left(t\right)}{dt}=k_{3}C_{\text{EES}}\left(t\right)-k_{4}C_{\text{EIS}}\left(t\right), \end{array}$$

where *C*_*p*_, *C*_EES_, and *C*_EIS_ are the concentrations of the tracer in the blood plasma, extravascular-extracellular, and extravascular-intracellular spaces, respectively. There are four unknown rate constants and three unknown concentration-of-tracer time courses. The problem is compounded by the fact that a typical PET study measures only the total concentration of the tracer in a given voxel or region of interest, *C*_tissue_, which is determined by the concentration of the tracer in each compartment and the relative volume contributions of each compartment:

(2) $$\begin{array}{c} C_{\text{tissue}}\left(t\right)=v_{b}C_{b}\left(t\right)+v_{\text{EES}}C_{\text{EES}}\left(t\right)+v_{\text{EIS}}C_{\text{EIS}}\left(t\right), \end{array}$$

where *v*_*b*_, *v*_EES_, and *v*_EIS_ are the blood, extravascular-extracellular, and extravascular-intracellular volume fractions, respectively. Solving the second two relations in (1) yields

(3) $$\begin{array}{c} C_{\text{EES}}\left(t\right)=\frac{K_{1}}{\alpha_{2}-\alpha_{1}}C_{p}\left(t\right)⊗\left[\left(k_{4}-\alpha_{1}\right)e^{-\alpha_{1}t}+\left(\alpha_{2}-k_{4}\right)e^{-\alpha_{2}t}\right],\\ C_{\text{EIS}}\left(t\right)=\frac{K_{1}k_{3}}{\alpha_{2}-\alpha_{1}}C_{p}\left(t\right)⊗\left[e^{-\alpha_{1}t}-e^{-\alpha_{2}t}\right],\\ \alpha_{1,2}=\frac{\left(k_{2}+k_{3}+k_{4}\right)\pm \sqrt{{\left(k_{2}+k_{3}+k_{4}\right)}^{2}-4k_{2}k_{4}}}{2}. \end{array}$$

If we note that *v*_*b*_ + *v*_EES_ + *v*_EIS_ = 1, *v*_*p*_ = *v*_*b*_ · (1 − hematocrit) and assume that the plasma free fraction is 1, then the solution (i.e., (2) and (3)) has six unknown parameters and three unknown concentration-of-tracer time courses. If the arterial input function can be measured reliably, this is reduced to two unknown concentration time courses. After briefly introducing the relevant aspects of DCE-MRI modeling, we proceed to show how DCE data can constrain this PET model by eliminating unknown parameters and determining unknown concentration-of-tracer time courses.

### 2.2. DCE-MRI Kinetic Modeling

 A typical DCE-MRI study employs an untargeted contrast agent that is distributed from blood into tissue, but is unable to appreciably penetrate cells, so the compartmental model is considerably simpler than the above model for PET tracer kinetics and is given as:

(4) $$\begin{array}{c} \frac{{dC}_{\text{tissue}}\left(t\right)}{dt}=K^{\mathrm{trans}}C_{p}\left(t\right)-\frac{K^{\mathrm{trans}}}{v_{\text{EES}}}C_{\text{tissue}}\left(t\right), \end{array}$$

where *C*_tissue_ and *C*_*p*_ are the concentration of an MRI contrast agent in the tissue and plasma space, respectively, *K* ^trans⁡^ represents the volume transfer constant for the agent between the blood plasma (in units of mL  (blood)/mL (tissue)/min) and the extravascular-extracellular space, and *v*_EES_ are as above. This corresponds to the first two compartments in Figure 1. The intracellular compartment concentration *C*_EIS_, along with the rate constants *k*_3_ and *k*_4_, is zero since standard, clinically approved MRI contrast agents remain extracellular. The solution to (4) is given as follows:

(5) $$\begin{array}{c} C_{\text{tissue}}\left(t\right)=K^{\mathrm{trans}}\exp\left(\frac{-K^{\mathrm{trans}}t}{v_{\text{EES}}}\right)⊗C_{p}\left(t\right). \end{array}$$

Many have noted that this model does not explicitly account for the plasma fraction, and thus (5) is frequently amended to include a blood plasma component as follows:

(6) $$\begin{array}{c} C_{\text{tissue}}\left(t\right)=K^{\mathrm{trans}}\exp\left(\frac{-K^{\mathrm{trans}}t}{v_{\text{EES}}}\right)⊗C_{p}\left(t\right)+v_{p}C_{p}\left(t\right), \end{array}$$

where *v*_*p*_ is the blood plasma fraction. By measuring heavily T1-weighted DCE-MRI data in the tissue of interest (e.g., a tumor) and a feeding vessel before, during, and after the injection of a standard extracellular contrast agent, the *C*_tissue_(*t*) and *C*_*p*_(*t*) time courses can be estimated and fit to (6) to extract *K* ^trans⁡^, *v*_EES_, and *v*_*p*_; the latter two can be used to assign *v*_EES_, *v*_*b*_ ( = *v*_*p*_/(1 − hematocrit)), and *v*_EIS_ ( = 1 − *v*_*b*_ − *v*_EES_) in the PET model.

### 2.3. Constraining PET Kinetic Modeling

 We now show how DCE-MRI data can be used to eliminate a number of the unknown quantities in (2) and (3). The method adapts the approach developed by Asllani et al. [4] for partial volume corrections in MRI studies of blood flow. 

 If we take the *v*_*p*_ ( = *v*_*b*_ · (1 − hematocrit)) and *v*_EES_ values returned from a typical DCE-MRI study as *a priori *knowledge for the PET analysis, then this reduces the number of unknowns to four rate constants (*K*_1_–*k*_4_) and three concentration time courses (*C*_*p*_, *C*_EES_, and *C*_EIS_). We then consider a DCE-MRI scan and a PET study that have been spatially registered such that they have the same voxel sizes. This implementation differs from the one given in [4] which does not incorporate spatial registration; we return to this point in Section 4. Next we take advantage of the differences in spatial resolution between the acquired MRI and PET data and define a small region of tissue, a 5 × 5 voxel window centered on a particular voxel of interest, from both the DCE-MRI and PET studies. If we assume that the tissue PET tracer concentration (the left hand side of (2)) in each voxel of the 5 × 5 window is equal to the *measured* tissue concentration in the central (particular) PET voxel and assume that the *C*_*b*_, *C*_EES_, and *C*_EIS_ are identical in each MRI voxel—reasonable assumptions for a small window—then we can write a system of equations in the three unknowns. In particular, for the *i*th voxel within the window, we have

(7) $$\begin{array}{c} C_{\text{tissue}}\left(t\right)=v_{i,b}C_{b}\left(t\right)+v_{i,\text{EES}}C_{\text{EES}}\left(t\right)+v_{i,\text{EIS}}C_{\text{EIS}}\left(t\right), \end{array}$$

where *i* runs from 1 to *N*, the number of voxels in the search window. If we define the matrix *A* to be

(8) $$\begin{array}{c} A=\left\lfloor \begin{array}{ccc} v_{1,b} & v_{1,\text{EES}} & v_{1,\text{EIS}}\\ v_{2,b} & v_{2,\text{EES}} & v_{2,\text{EIS}}\\ \cdots & \cdots & \cdots \\ v_{N,b} & v_{N,\text{EES}} & v_{N,\text{EIS}} \end{array}\right\rfloor \end{array}$$

and the column vector *C* to be

(9) $$\begin{array}{c} C=\left[\begin{array}{c} C_{b}\\ C_{\text{EES}}\\ C_{\text{EIS}} \end{array}\right], \end{array}$$

then we can rewrite the system in (7) as

(10) $$\begin{array}{c} C_{\text{tissue}}=A_{ij}C_{i}, \end{array}$$

where *C*_tissue_ is a column vector of length *N*. The optimal least-squares solution to (10), which is an overdetermined problem (5), is given by

(11) $$\begin{array}{c} C={\left(A^{T}\cdot A\right)}^{-1}A^{T}\cdot C_{\text{tissue}}, \end{array}$$

where (*A* ^*T*^ · *A* ^−1^)*A* ^*T*^ is the pseudoinverse and every term on the right-handside is known. In principle, this analysis should return time courses for *C*_*b*_ (and thus *C*_*p*_(*t*)), *C*_EES_, and *C*_EIS_, which can then be used to drive the PET kinetic analysis described by (3) to return the *k*_*i*_. This approach also solves the problem of not knowing how to separate the measured whole tissue PET signal into *C*_EES_, *C*_EIS_, and *C*_*p*_ components.

### 2.4. Simulations

 To evaluate the theory above, we simulated kinetic PET data over a range of parameter combinations. The simulations were initialized with an arterial input function *C*_*p*_(*t*) measured from a dynamic ^18^F-fluorothymidine (FLT) PET scan (data not shown as the particular tracer employed is not central to this paper; that is, all that is needed is a reasonable, experimentally measured input function). This time course was then used to drive (2)-(3) with *k*_*i*_ values that were randomly selected from uniform distributions. The range of the distribution of the parameters used here was chosen to be of the same order as those given in the literature [5, 6], though we again note that the parameters listed in (2)-(3) are not the same as those in the references. Experimental noise was simulated in two ways. For each time point, the standard deviation of the noise level was set to 1, 10, 20, 30, 40, and 50 times the square root of the whole tissue concentration simulated at that time point [7]. A second source of systematic error was included by varying the accuracy of the parameters extracted from the DCE-MRI analysis and used as *a priori* data for (11). As indicated by (8), 25 sets of values of *v*_*b*_, *v*_EES_, and *v*_EIS_ are required per PET voxel. Reliable values are available for *v*_*b*_ and *v*_EES_ so we assigned the 25 voxel values randomly selected from uniform distributions with ranges from 0.04 to 0.12 and 0.25 to 0.45, respectively [8–10]. Each *v*_EIS_ value was assigned according to *v*_EIS_ = 1 − (*v*_*b*_ + *v*_EES_). For each noise realization, the simulated whole tissue data were first analyzed *via* (11) to extract estimates of the *C*_EES_, *C*_EIS_, and *C*_*b*_ time courses. After the first step, we computed the concordance correlation coefficient (CCC) between the extracted *C*_EES_, *C*_EIS_, and *C*_*b*_ and the true *C*_EES_, *C*_EIS_, and *C*_*b*_ time courses. The CCC tests the strength of the correlation, as well as the deviation of the correlation from the line of unity. The extracted time courses were then analyzed with (2) and (3) to return estimates of the kinetic PET parameters. While there are several ways to execute this fitting, we elected to perform a procedure whereby *C*_*b*_ and *C*_EES_ were combined with the first expression in (3), *C*_*b*_ and *C*_EIS_ were combined with the second expression in (3), and the residuals for both sets of data were optimized simultaneously. This procedure was performed 1000 times with different combinations of parameters and realizations of noise, and the extracted PET parameters were then compared to their actual values and the mean ± the 95% confidence interval was computed. We then performed a second set of simulations to examine the effect of error in the DCE-MRI parameters on the accuracy of the kinetic PET parameters. The noise level in the simulated TACs was set to the square root of the whole tissue concentration and an error was added to the DCE-MRI parameters such that the 95% confidence interval of the assigned parameter value was 0%, 5%, 10%, and 15% of the mean value. Higher errors in the DCE-MRI parameters yielded unreliable results in the PET kinetic analysis. As before, this procedure was performed 1000 times and both the CCC values between the true and extracted time courses and the error in the extracted PET parameters were computed.

## 3. Results

**Figure 2:**
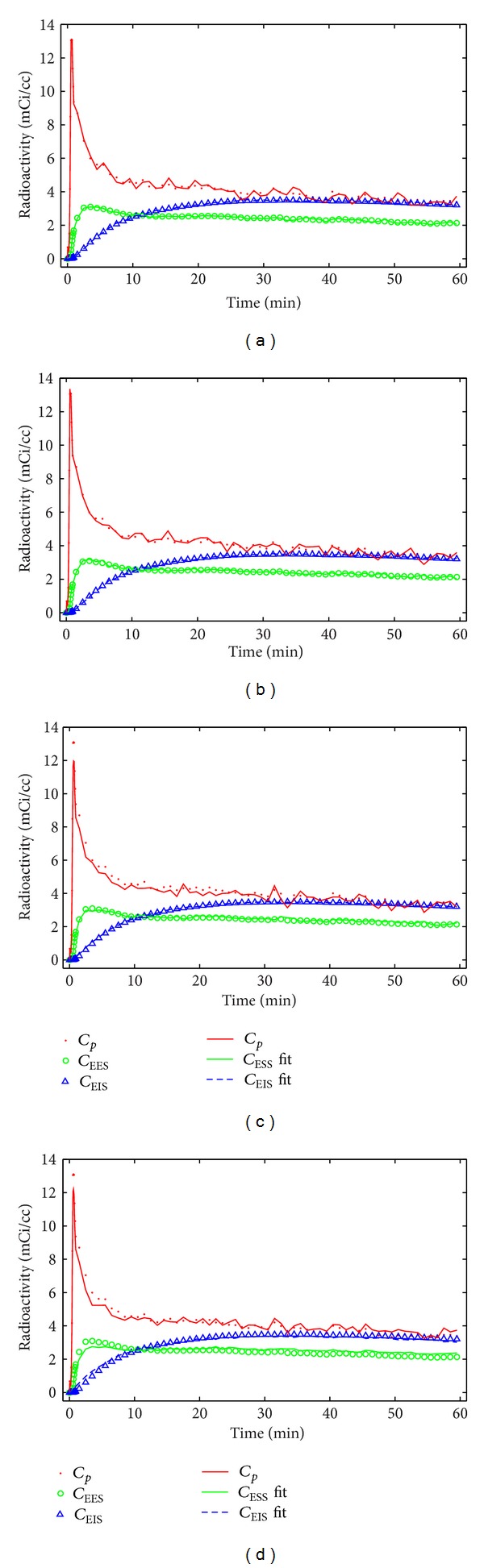
An example of simulated tissue curves and the fits provided by (11). These panels represent 25 voxels with (a) no error in the DCE-MRI parameters, (b) 5% error, (c) 10% error, and (d) 15% error. The AIF used in these simulations was measured from the left ventricle of a mouse, which results in some noise even with no error in the DCE-MRI parameters (see panel (a)).

Figure 2 shows the ability of the algorithm to correctly separate the *C*_EES_, *C*_EIS_, and *C*_*b*_ time courses from one simulated, whole tissue dataset. The parameter values were *K*_1_ = 0.3 (mL/min/g), *k*_2_ = 0.5 (min^−1^), *k*_3_ = 0.15 (min^−1^), and *k*_4_ = 0.1 (min^−1^). The solid lines indicate the extracted curves, while the individual points correspond to the true (simulated) data; the filled circles in each panel depict the measured *C*_*b*_ time course used to drive all the simulations. The four panels correspond to time courses extracted when the DCE-MRI parameters have errors of 0%, 5%, 10%, and 15%. In all four panels, the error in *C*_EIS_ is less than 5%. *C*_EES_ is extracted with an error of less than 5% when the DCE-MRI parameters have errors of 10% or less (panels (a)–(c)). The maximum error in *C*_EES_ when the DCE-MRI parameters have errors of 15% is approximately 10%. For the *C*_*b*_ component, the maximum error in the extracted time course increases with the errors in the DCE-MRI parameters.

**Figure 3:**
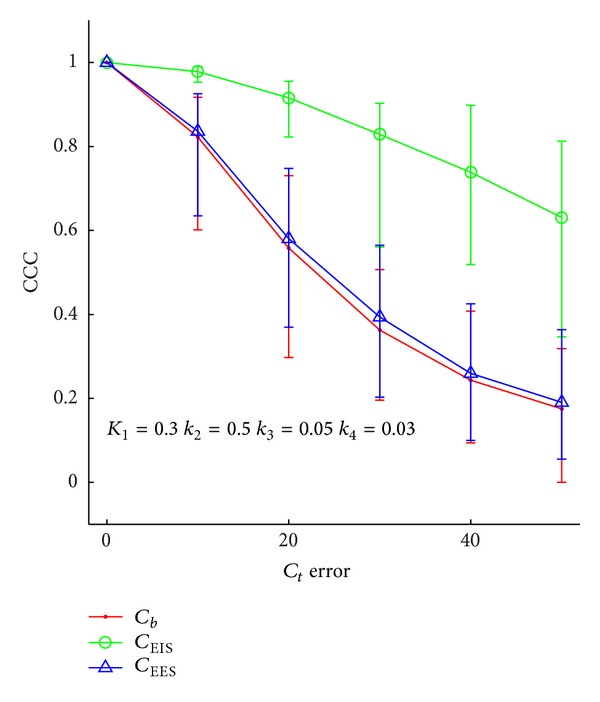
The concordance correlation coefficient between the estimated and true values of the time courses as a function of noise in the tissue curves for a single set of PET kinetic parameters. If the *C*_*t*_ error is less than 10 times the square root of activity level, then the CCC is greater than 0.75. Once *C*_*t*_ error is greater than this, the ability to faithfully reconstruct the time courses is substantially reduced.

**Figure 4:**
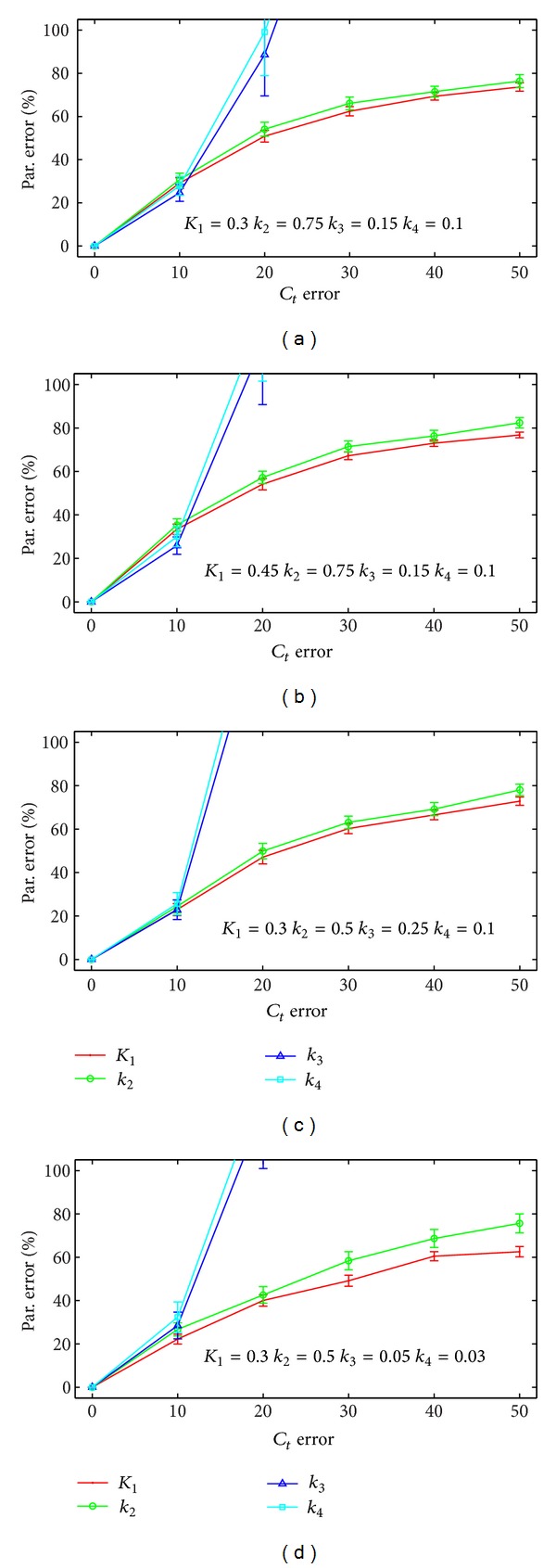
The error in the estimated PET kinetic parameters as a function of the noise in the tissue curves. Each panel corresponds to a different set of PET kinetic parameters. For each set of parameters, when the error in *C*_*t*_ is less than 10 times the square root of the activity level, the parameter error is less than 50. As the error in *C*_*t*_ continues to increase, the error in *k*_3_ and *k*_4_ increases rapidly. The error in *K*_1_ and *k*_2_ also continues to increase, but less rapidly.

 As stated above, two sets of noise realizations were performed. Figures 3 and 4 display results of simulations done with varying levels of noise added to the simulated *C*_tissue_ curves, and with no error in the DCE-MRI parameters.  Figure 3 shows the CCC (vertical axis) between the extracted and true *C*_EES_, *C*_EIS_, and *C*_*b*_ time courses as a function of increasing *C*_tissue_ noise for one combination of PET kinetic parameters. The results are presented as the mean ± the minimum and maximum CCC values obtained over the 1000 noise realizations. The results from the other seven combinations of PET kinetic parameters are similar. In all cases, when no noise is added to the simulated *C*_tissue_ curves, the CCC values are uniformly 1.00. The mean CCC values decrease with increased noise, with the CCC for *C*_*b*_ and *C*_EES_ decreasing to 0.2 at the maximum level of noise tested here. For all combinations of PET kinetic parameters, the *C*_EIS_ time courses are the least affected by noise in the tissue curves.

Figure 4 presents four panels showing the error in the extracted *k*_*i*_ as a function of the error in the DCE-MRI kinetic parameters. Each panel corresponds to a different combination of *k*_*i*_ values that are listed at the bottom of each panel. The data are presented as mean ± one standard error. The error trends in the other sets of PET kinetic parameters is similar to those shown. Across all parameter combinations, the error in the extracted PET kinetic parameters is below 50% provided that the error in the simulated *C*_*t*_ curves is below 20 times the square root of the concentration. However, as the noise continues to increase, the error in the *k*_3_ and *k*_4_ parameters increases rapidly to above 100%. The error in *K*_1_ and *k*_2_ parameters increases less rapidly than that for *k*_3_ and *k*_4_.

**Figure 5:**
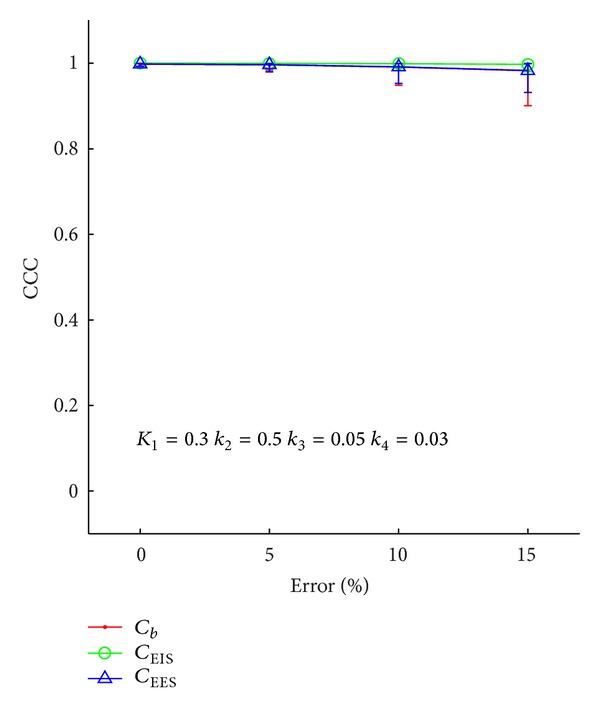
The concordance correlation coefficient between the estimated and true values for the time courses as a function of error in the DCE-MRI parameters for a single set of PET kinetic parameters. The method is able to return the time courses faithfully when the DCE-MRI parameter error is less than 5%. With higher error in the DCE-MRI parameters, the CCC remains above 0.95 on average, though some realizations returned CCC values as low as 0.9.

**Figure 6:**
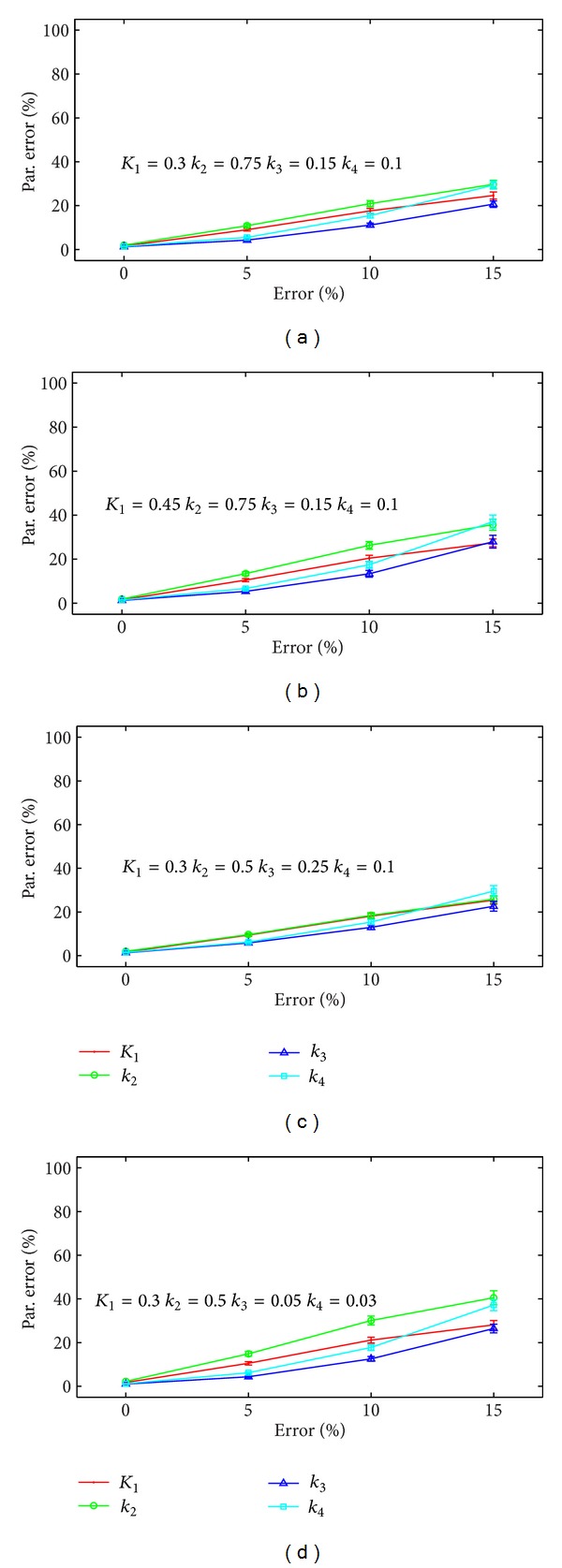
The error in the estimated PET kinetic parameters as a function of the error in the DCE-MRI parameters. Each panel corresponds to a different set of PET kinetic parameters. For each parameter combination, the error in the PET parameters is below 25% provided the error in the DCE-MRI parameters is below 10%. As the DCE-MRI parameter error increases, the error in the PET parameters exceeds 40% in some cases (see panel (d)).

 Figures 5 and 6 display similar results as those in Figures 3 and 4. These two figures display the results from the simulations where the error in the DCE-MRI parameters increased from 0 to 15%. As stated above, in these simulations, the error in the *C*_*t*_ time course was fixed to the square root of the concentration in the tissue.  Figure 5 shows the CCC between the extracted and true *C*_EES_, *C*_EIS_, and *C*_*b*_ time courses as a function of percent error in the DCE-MRI parameters for a single set of PET kinetic parameters. The mean CCCs are all above 0.9 across all parameter combinations and noise realizations. The minimum CCC over all the realizations was 0.6, which occurred when the error in the DCE parameters is 15%. 

Figure 6 again presents four panels showing the error in the extracted *k*_*i*_ as a function of the error in the DCE-MRI kinetic parameters. The range and combinations of the *k*_*i*_ are the same as in Figure 5. The data are presented as mean ± one standard error. Across all eight parameter combinations tested, the errors in the extracted PET kinetic parameters are below 25% provided that the error in the DCE-MRI parameters is below 10%. However, once the DCE-MRI error approaches 15%, there are certain combinations of kinetic PET parameters that lead to errors as high as 50% in the extracted parameters; one example can be seen in panel (d).

## 4. Discussion

Since the advent of the first prototype SPECT-CT system in 1990 [11, 12], multimodality imaging has been largely focused on combining form and function. In particular, SPECT-CT and PET-CT systems have combined the high-resolution anatomical images available through CT imaging with the molecular information provided by nuclear medicine [13, 14]. New developments in multimodality imaging are increasingly focused on combining data sets to enable new measurements not previously possible. One example of this new type of functional imaging is the combination of dynamic PET imaging with dynamic MR imaging. The introduction of PET/MR hybrid scanners [15–17] makes the approach outlined here practical. With these scanners, the data could be acquired at nearly the same time and would be readily registered. 

The results from the simulations show that the method returns good estimates for the time courses *C*_*b*_, *C*_EES_, and *C*_EIS_ as well as the PET kinetic parameters *k*_*i*_ when the noise in the tissue curves and the error in the volume fractions measured by DCE-MRI are both small. The method is highly sensitive to increased noise in the TACs, as seen in Figures 3 and 4. This is a potential limitation of the proposed method. More sophisticated imaging or postprocessing techniques focusing on reducing the noise in the measured PET data would allow for higher accuracy in the estimated PET kinetic parameters.

As seen in Figures 5 and 6, the accuracy of the PET parameters also depends on how well the DCE-MRI parameters are known. The dependence of the PET parameter estimates on the error in the DCE-MRI parameters is smaller than the dependence on the noise in the tissue concentration curves. Other studies have shown that the error in the DCE-MRI parameters depends on several factors, including the accuracy of the arterial input function used to estimate the parameters [18, 19]. Methods for obtaining higher accuracy in DCE-MRI parameters are an area of active research (see, e.g., [20, 21]). Recent work assessing the reproducibility of DCE-MRI parameters in preclinical models of breast cancer reported that parameter measurements for *v*_EES_ are repeatable between imaging sessions [22], which suggests that, with no systemic modeling errors, obtaining *v*_EES_ measurements with small errors is possible. The repeatability index for *v*_*b*_ was smaller and may be more susceptible to measurement error. Similar work in clinical applications of DCE-MRI also reported high repeatability for *v*_EES_ [23, 24].

 The method developed by Asllani et al. [4] took advantage of the difference in spatial resolution between echo planar and arterial spin labeled MRI acquisitions to develop a system of equations similar to that given previously in (7). In that implementation, the echo planar images and arterial spin-labelled images were acquired sequentially on the same system and thus were inherently coregistered. In our method, the PET and MR images would typically be acquired on different scanners (though, as noted above, PET-MRI scanners are becoming more commonplace), and some spatial registration would be required prior to image processing. As mentioned above, our approach assumes that the TACs in each compartment are identical within a 5 × 5 window of MRI voxels, which is reasonable for MRI voxel sizes of approximately 250 *μ*m × 250 *μ*m in-plane resolution which is common for *in vivo* DCE-MRI studies in mice.

 As noted in the Introduction, the parameters *k*_*i*_ used in this formulation have a fundamentally different meaning than those commonly employed in PET kinetic modeling. Typically, compartments used in dynamic PET modeling are based on biochemical compartments, whereas in dynamic MRI modeling they are based on physical compartments. One consequence of this difference is that standard metabolic rate constants cannot be calculated directly from the formalism as presented in this effort. However, even though the rate constants calculated from this model do not reflect the biochemical state of the radiotracer, they do provide information on the physical movement of radiotracers into and out of the vascular and cellular spaces. This set of parameters is potentially useful in situations where the physical location of the tracer (i.e., intra- or extracellular) may be relevant to patient diagnosis or treatment planning. For example, this model may be of potential interest in measuring the cellular uptake of glucose in dynamic FDG studies in diabetic populations. The new *k*_3_ and *k*_4_ parameters returned by this model reflect the rate of transport of tracer into and out of the intracellular space, respectively. These parameters may be able to report on GLUT-1 and GLUT-3 transportation in tumor cells. This model could also be of potential interest in dynamic [^15^O]-labeled water studies. Typical modeling of [^15^O]-labeled water utilizes a two-compartment model with one intravascular compartment and one extravascular compartment [25]. This is due to difficulty in resolving intra- and extracellular compartments due to the free diffusion of water across the cell boundary. Using DCE-MRI data to inform the fractional volume of these compartments may provide further insight into the molecular physiology. Of course, the preclinical and clinical utility of the “new” *k*_*i*_ parameters is left to future work. 

 It should also be noted that performing the analysis described in this work would, of course, not preclude any dynamic modeling with more traditional compartment definitions on the same data. Also, despite the changes in the physical interpretation of the rate constants, the arterial input function derived from the proposed method is identical to that used in more traditional dynamic PET modeling and can be used in implementing these models. Perhaps, the method proposed in this effort has value in merely providing an input function from which a standard dynamic analysis could be performed. Additionally, the input function estimated by this approach comes from the (local) tissue of interest which could potentially eliminate uncertainties related to the delay and dispersion when an input function is estimated from blood samples or ROIs in distant locations.

 Future studies with this method will focus on validating the proposed method with *in vivo* data. Higher temporal resolution in the DCE-MRI data may allow for improved accuracy in the DCE-MRI parameters, but acquiring this data would cost some spatial resolution. The noise in the PET data could also be improved by effectively lowering the spatial resolution through averaging the data. The proposed method may be more effective in situations where the desire for quantitative information outweighs the need for high spatial resolution information.

## 5. Conclusion

We have presented a method that uses DCE-MRI parameters to separate the whole tissue concentration curves, *C*_tissue_, into extravascular-extracellular, extravascular-intracellular, and intravascular components. These separate components are then used to initialize and constrain the model fitting for a dynamic PET compartment model. We show in simulation that this method returns PET parameters with less than 25% error provided that the noise in the tissue curves and the error in the DCE-MRI parameters are both small. In the limit of perfectly known DCE-MRI parameters and no noise in the tissue curves, the method returns parameters with no error. These preliminary (theoretical) results warrant *in vivo* experimental studies to validate or refute the method.
